# Neural networks underlying contributions from semantics in reading aloud

**DOI:** 10.3389/fnhum.2013.00518

**Published:** 2013-09-02

**Authors:** Olga Boukrina, William W. Graves

**Affiliations:** Department of Psychology, Rutgers, The State University of New JerseyNewark, NJ, USA

**Keywords:** semantics, effective connectivity, reading, fMRI, phonology, orthography

## Abstract

Reading is an essential part of contemporary society, yet much is still unknown about the physiological underpinnings of its information processing components. Two influential cognitive models of reading, the connectionist and dual-route cascaded models, offer very different accounts, yet evidence for one or the other remains equivocal. These models differ in several ways, including the role of semantics (word meaning) in mapping spelling to sound. We used a new effective connectivity algorithm, IMaGES, to provide a network-level perspective on these network-level models. Left hemisphere regions of interest were defined based on main effects in functional magnetic resonance imaging and included two regions linked with semantic processing—angular gyrus (AG) and inferior temporal sulcus (ITS)—and two regions linked with phonological processing—posterior superior temporal gyrus (pSTG) and posterior middle temporal gyrus (pMTG). Participants read aloud words of high or low spelling-sound consistency, word frequency, and imageability. Only the connectionist model predicted increased contributions from semantic areas with those computing phonology for low-consistency words. Effective connectivity analyses revealed that areas supporting semantic processing (e.g., the ITS) interacted with phonological areas (e.g., the pSTG), with the pattern changing as a function of word properties. Connectivity from semantic to phonological areas emerged for high- compared to low-imageability words, and a similar pattern emerged for low-consistency words, though only under certain conditions. Analyses of individual differences also showed that variation in the strength of modulation of ITS by AG was associated with reading aloud performance. Overall, these results suggest that connections with semantic processing areas are not only associated with reading aloud, but that these connections are also associated with optimal reading performance.

## Introduction

The ability to process written language is fundamental to our capacity to encode and transmit the wealth of human knowledge. In contemporary society where text is ubiquitous, reading deficits represent a significant handicap. Yet, despite decades of research into the cognitive and neural mechanisms of reading, several basic questions remain unresolved, such as whether there are multiple routes to reading or a single basic system and whether word meaning plays a role in reading aloud.

Cognitive theories of reading differ with respect to the role that access to meaning (semantics) is thought to play during conversion of spelling into sound. According to single-process connectionist models (e.g., Seidenberg and McClelland, 1989; Plaut et al., 1996), orthography-phonology (orth-phon) mapping develops according to the frequency of exposure to spelling-sound correspondence patterns. This process is mediated by semantics, with the amount of semantic input depending on the nature of the word. Words with consistent spelling-sound correspondence patterns (e.g., UST in DUST) can be pronounced without strong activation of semantic (sem) content, whereas words with inconsistent spelling-sound correspondence patterns (e.g., OST in HOST and COST) rely on access to meaning to a greater degree. In this model, semantics is used to reduce interference between inconsistent features of the input.

In contrast to connectionist models, the dual-route cascaded model (DRC; e.g., Coltheart et al., 1993, 2001) poses that semantic properties do not affect the early stages of word processing. Written words are processed in parallel along two-routes: a *direct* look-up in the orth and phon lexicons, or an *indirect* orth-phon conversion. The direct lexical route can be further subdivided into the lexical non-semantic and lexical-semantic pathways, but semantics has not been fully implemented in this model. Although Coltheart et al. (1999) described an implementation of a 3 word lexicon to simulate the Stroop effect, to our knowledge this work has not advanced further. Moreover, any semantic activation in the direct pathway is assumed to be “too slow to influence skilled word pronunciation” (Plaut et al., 1996, p. 60). According to the DRC model, both the direct and the indirect route are engaged during reading aloud; however, for irregular words like YACHT, only the direct route will produce the correct pronunciation. Regular words like SHIP, on the other hand, rely to a considerable degree on processing in the indirect route, where high spelling-sound regularity of such words allows mapping from orth to phon using a set of correspondence rules (Coltheart et al., 1993, 2001).

The two models propose different processing mechanisms for reading aloud, which we infer should rely on different neural substrates. This allows us to test them using neuroimaging data. A recent meta-analysis of neuroimaging studies of reading suggested that localization evidence can help constrain these cognitive models by offering information about functional overlap or separation of lexical processes in the brain (Taylor et al., 2013). The Taylor et al. analysis represents an important step in bringing functional neuroimaging data to bear on models of reading in a systematic way. The authors stated that their primary goal was not to adjudicate between connectionist and dual-route models of reading, and there were at least two factors that would have hindered their ability to do so. The first was that in order to have sufficient data for an effective meta analysis, they included studies using a variety of reading-related tasks, not only reading aloud but also silent reading, lexical decision, visual feature detection, etc. This variability produced uncertainty regarding the functions ascribed to specific brain regions. For example, the meta-analysis showed that the words > pseudowords contrast was associated with activations in the left anterior fusiform, middle temporal and angular gyri, the putative locations of the orthographic and phonological lexicons proposed as part of the dual-route model (Coltheart et al., 2001). Yet given the overlap of these activations with the Binder et al. (2009) meta-analysis, which applied very strict selection criteria for studies of semantic processing, these brain activations may instead correspond to the computation of word meaning. Such a result would be consistent with the connectionist model. Here we focus on reading aloud because, as Taylor et al. (2013) acknowledge, the model predictions diverge in terms of whether or not they propose a role for semantics in reading aloud. Such a focus would not have been possible in a meta-analysis given the limited number of studies using reading aloud in functional neuroimaging.

**Figure 1:**
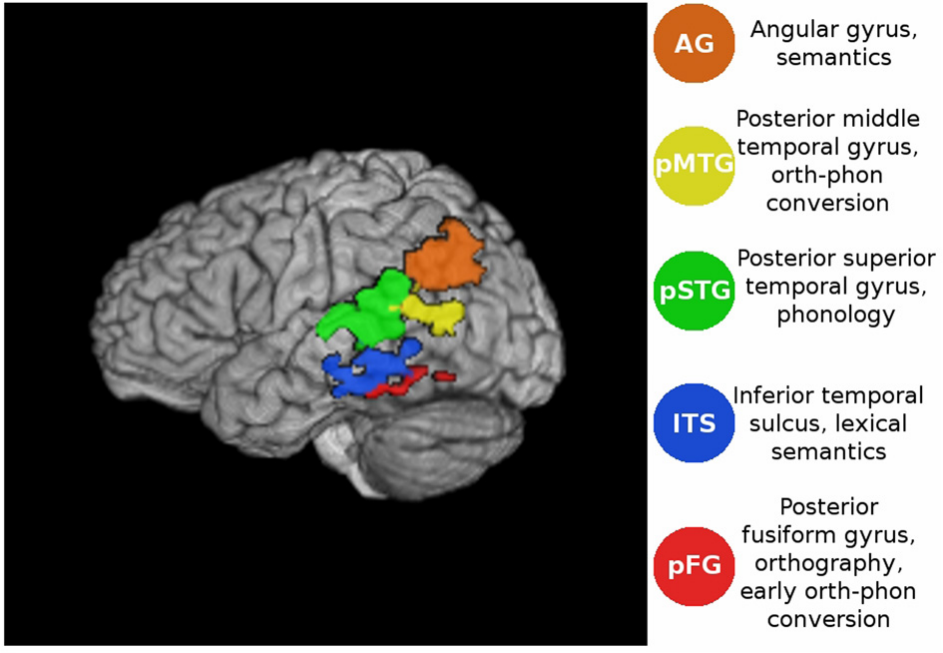
**Volume rendering of the functional ROIs in the lateral view of the atlas brain.** For coordinates see Table 1.

In accordance with the theoretical assumptions of the single-process model, in this study we expected that variations in word-frequency, spelling-sound consistency, and imageability would produce different patterns of effective connectivity among the 5 regions of interest (ROIs) shown in Figure 1 and described below. We were particularly interested in the division of labor between the phonological and the semantic pathways and the predictions that stem from the relative contribution of each pathway to reading aloud. If, for example, semantic access plays a role in reading aloud, then different patterns of network connectivity would be expected when reading words that differ in how much they engage the semantic system through a factor such as imageability (e.g., Strain et al., 1995). Imageability ratings reflect the relative ease with which a word evokes an image. Highly imageable words have richer, more easily computed semantic representations than less imageable words (Paivio, 1991; Schwanenflugel, 1991; Plaut and Shallice, 1993). Such words would be expected to rely to a greater degree on an interaction between regions involved in semantic processing and regions linked with orthographic and phonological processing. Similarly, words with low spelling-sound consistency may require additional involvement of the semantic system to help map their orthography to the correct pronunciation. By associating orth, phon and sem processes with different parts of the reading network, we can make predictions about the connectivity patterns that will be obtained under the assumptions of competing cognitive models.

**Table 1:** **Volumes (in mm^3^) and center of mass coordinates (in MNI space) for ROIs shown in Figure 1**.

**Label**	**Volume**	**X**	**Y**	**Z**
pSTG	5305	−50	−37	8
AG	4017	−45	−64	27
ITS	1854	−55	−37	−15
pMTG	1318	−50	−60	7
pFG	447	−38	−45	−20

pSTG, posterior superior temporal gyrus; AG, angular gyrus; ITS, inferior temporal sulcus; pMTG, posterior middle temporal gyrus; pFG, posterior fusiform gyrus.

The connectionist dynamics of reading aloud have been described in detail by Plaut et al. (1996). During typical reading the semantic pathway provides additional input to the phoneme units pushing them to their correct levels of activation. This additional input from semantics alleviates the pressure for the phonological pathway to master all the pronunciations. When the model is trained extensively, and the semantic pathway gains competence, the phonological pathway becomes increasingly specialized for reading consistent words, at the expense of inconsistent ones. Unlike in the dual-route model, however, even with extensive training the phonological route of the connectionist model can read inconsistent words, particularly those of high-frequency. Patient work has shown that when the semantic pathway is impaired the severity of reading disability is correlated with the amount of semantic deterioration (e.g., Patterson and Hodges, 1992). Similarly, as the amount of semantic input to the model's phoneme units is reduced (by degrading the semantic units), a gradual pattern of reading impairments emerges. Performance on the low-frequency inconsistent words is affected first, followed by high-frequency inconsistent words. If semantic input is completely eliminated, performance on the low-frequency consistent words is also affected (Figure 25, p. 98 of Plaut et al., 1996). These findings support the notion that normal reading is accomplished via a division of labor between the phonological and the semantic pathways and that neither of them is completely competent in isolation. Following this logic, we expected that the amount of semantic input would vary depending on the nature of the words.

A detailed connectionist investigation of the role of semantic information in single-word reading was performed by Harm and Seidenberg (2004). They studied reading for meaning, where orthography mapped to semantics either directly or through phonology. Although they explicitly acknowledge that the network dynamics for reading aloud will be different than reading for meaning, consistency effects were greater for the orth→phon→sem pathway than the orth→sem pathway (Harm and Seidenberg, 2004, p. 691). That is, consistent words were read more accurately than inconsistent words when the pathway involved phonological mapping, whereas inconsistent words benefitted from the essentially arbitrary orth→sem mapping. Because reading aloud involves mapping to phonology rather than stopping at semantics, we expect connections from semantic (ITS, AG) to phonological regions (pSTG, pMTG) to emerge for words that do not have consistent orth→phon mappings^1^.

Using a previously collected fMRI dataset (Graves et al., 2010), we examined patterns of effective connectivity for words that differed in imageability, consistency, and frequency. Functional ROIs were defined in regions that Graves et al. found to be sensitive to lexical characteristics such as imageability [angular gyrus (AG), orth-phon consistency inferior temporal sulcus (ITS), posterior fusiform gyrus (pFG)], bigram frequency [posterior middle temporal gyrus (MTG), posterior superior temporal gyrus (pSTG)], and word frequency (pFG, AG). Other neuroimaging studies have also shown that a similar set of regions reliably activates during reading. For example, the orthographic composition of a visually presented word is thought to be analyzed along the length of the left FG, with increasingly complex elements processed in more anterior parts of the FG (Vinckier et al., 2007). The middle portion of the FG is often referred to as the Visual Word Form Area (VWFA), due to its association with orthographic processing (e.g., Cohen et al., 2000, 2002; Dehaene et al., 2004; Binder et al., 2006). Neural computation of phonology is linked with activity in peri-Sylvian regions such as pSTG (e.g., Hickok and Poeppel, 2004; Graves et al., 2007, 2008; Price, 2012) and orth-phon conversion is associated with activity in pMTG (Jobard et al., 2003; Sandak et al., 2004), as well as other regions, such as SMG (Jobard et al., 2003; Sandak et al., 2004; Katz et al., 2005; Vigneau et al., 2006; Cattinelli et al., 2013) and opercular IFG (Pugh et al., 1996; Fiez and Petersen, 1998; Jobard et al., 2003; Hickok and Poeppel, 2004; Sandak et al., 2004; Katz et al., 2005). Finally, among the areas involved in processing semantics are the ITS (Binder et al., 2009), AG (Binder et al., 1997, 2005, 2009; Price and Mechelli, 2005; Price, 2012) and triangular IFG (Poldrack et al., 1999; Bookheimer, 2002; Jobard et al., 2003; Binder et al., 2009).

The 5 ROIs considered here were selected because they showed reliable activations across participants in our previous univariate analysis of this dataset (Graves et al., 2010), and had clear functional interpretations (see Figure 1) on which we also based a structural connectivity analysis (Graves et al., submitted). Under the connectionist account, effective connectivity graphs should exhibit increased engagement of the semantic system during reading of words with high imageability and low consistency. We also expected to find a frequency-by-consistency interaction (e.g., Paap and Noel, 1991), such that the differential contribution of semantics to reading low- and high-consistency words would be greatest when these words are also of low frequency. This should be most evident in areas previously implicated in semantic processing such as the ITS (Binder et al., 2009; Graves et al., 2010) and AG (Binder et al., 1997, 2005, 2009; Price and Mechelli, 2005; Price, 2012). Connectionist models also posit that phonology is assembled rather than accessed from a whole-form lexicon. Therefore, during reading aloud regions involved in orth→phon conversion, such as the pMTG (Jobard et al., 2003; Sandak et al., 2004; Graves et al., 2010), were expected to be co-activated with regions involved in phonological processing (pSTG; e.g., Hickok and Poeppel, 2004, 2007; Graves et al., 2007, 2008; Price, 2012). In cases where the mapping between orthography and phonology was highly consistent, particularly for stimulus words of low imageability, the engagement of semantic processing was expected to be minimal. In this case we expected to see a direct link from occipitotemporal orthographic regions to posterior MTG and STG for engagement of orthography-phonology conversion and phonological recoding prior to speech.

In addition to a differential contribution of semantics to processing high and low levels of word frequency and consistency, we expected that semantic processes may be differentially utilized by individual readers. Recently we showed that the effect of semantic variables on brain activity varied considerably across individuals and was correlated with the volume of the neural pathways that connect posterior temporal areas with inferior temporal and parietal regions, involved in access to meaning (Graves et al., submitted). Although the association between use of semantics in reading and structural connectivity among proficient readers was novel, previous studies have found neural associations with individual differences in other aspects of reading (e.g., Bolger et al., 2008; Seghier et al., 2008; Levy et al., 2009; Seghier and Price, 2009). Plaut et al. (1996) also argue that premorbid differences in the reliance on semantic support, stemming from differences in the nature of reading instruction, the quality of phonological representations, relative experience in reading aloud vs. silently, as well as general computational resources, may explain the differences in the level of reading impairment in patients who have comparable amounts of brain damage. To further investigate patterns of effective connectivity in the functional neural data, we tested for individual differences in these patterns with measures of the behavioral influence of three relevant stimulus properties: spelling-sound consistency imageability, and word frequency.

Recent advances in cognitive neuroscience have begun to make distributed, network-level analyses of functional brain imaging data tractable and reliable (Hanson and Halchenko, 2008; Poldrack et al., 2009; Ramsey et al., 2010, 2011). For example, the interactivity within neural systems has been examined using effective connectivity analyses (McIntosh and Gonzalez-Lima, 1994; Friston, 2003; Ramsey et al., 2010, 2011; Schuyler et al., 2010), comprised of algorithms that quantify the influence one brain region exerts on another and, consequently, can uncover the underlying causal structure of network activity. Such an approach should be capable of uncovering the underlying causal structure of network activity (Friston and Büchel, 2003). These analyses, combined with previous localization evidence, offer novel ways of investigating the neural dynamics of reading. They can help adjudicate between competing views by uncovering the patterns of neural interactions between areas supporting semantic, orthographic and phonological processes.

The success of a network-level investigation depends crucially on the use of a valid and reliable method of analysis to measure connectivity within the network. A troubling finding from a stimulation study by Smith et al. (2011) showed that of the 38 effective connectivity algorithms currently used in neuroimaging, none could find both connections and their orientations in 28 simulated networks without a large number of false positives, false negatives or both. However, Ramsey et al. (2011) showed that a graphical search algorithm, Independent Multiple sample Greedy Equivalence Search (IMaGES; Ramsey et al., 2010), combined with an orientation algorithm, Linear non-gaussian Orientation, Fixed Structure (LOFS), delivered high precision (>80%) in the reproduction of connections and orientations for all 28 simulated networks. The difficulty in arriving at a correct pattern of effective connectivity stems from computational intractability, which often arises because the number of alternative causal structures for a set of, say, 10 ROIs, could be on the order of billions (Ramsey et al., 2010). IMaGES solves this problem by using Bayesian methodology to partition the connectivity search space into manageable chunks of Markov equivalence classes and by further constraining the search to connections that carry the greatest predictive power (Perez et al., 2010). The advantages of this algorithm include greater flexibility to uncover new connectivity patterns not previously seen in reading studies, and the ability to provide better model fit. Here we used IMaGES analysis in concert with the theoretical approach of connectionist models to develop a network-level neural account of reading.

## Methods

### Participants

The participants were 20 (13 female) healthy, literate, right-handed volunteers with normal or corrected-to-normal vision. The mean age of participants at the time of the study was 23.2 (*SD* = 3.4). All participants provided written informed consent before taking part in the reading aloud fMRI study as described in Graves et al. (2010).

### Materials

The stimuli were 465 monosyllabic English words selected such that their length, log-transformed frequency of occurrence, spelling-sound consistency, imageability, and log-transformed position-constrained bigram and biphone frequencies were uncorrelated. A detailed description of the stimuli is available in Graves et al. (2010).

### Task and data acquisition

The experiment used a fast event-related fMRI design with continuous acquisition. On each trial a word appeared on the screen for 1000 ms before being replaced by a fixation cross of variable duration, with a mean intertrial interval of 4.9 s (*SD* = 3.72). Participants' task was to “read each word aloud as quickly and accurately as possible” and their responses were recorded with an MRI-compatible microphone.

A 3.0-T GE Excite (GE Healthcare, Waukesha, WI) MRI scanner with an 8-channel array radio frequency head coil was used for data acquisition. Functional images were acquired using a gradient-echo echoplanar imaging (EPI) sequence (*TE* = 25 ms; *TR* = 2000 ms; FOV = 192 mm; matrix = 64 × 64). Thirty-two interleaved axial slices per volume were obtained (3 × 3 × 2.5 mm voxels, 0.5 mm gap). The data were acquired in 5 functional runs with 240 whole-brain image volumes each. High resolution, T1-weighted anatomical images were acquired using a spoiled-gradient-echo sequence (matrix = 0.938 × 0.938 mm; 134 contiguous 1 mm axial slices).

### fMRI data analysis

Image preprocessing was performed using FSL 5.0 software (FMIRB's Software Library, www.fmirb.ox.ac.uk/fsl). Functional images were skull stripped using BET (Smith, 2002) and registered to high-resolution anatomical and standard MNI (Montreal Neurological Institute) space images using FLIRT (Jenkinson and Smith, 2001; Jenkinson et al., 2002).

Mean activation timeseries were extracted from each participant's registered and skull-stripped fMRI data and each ROI. The ROIs were defined in MNI space (Grabner et al., 2006) from previous fMRI results (Graves et al., 2010) taken directly from that study using the exact significance and extent criteria described previously. The only modifications made were to apply anatomical masks so the regions did not extend beyond relevant anatomical boundaries, as defined in the Talairach atlas (Lancaster et al., 2000). This ensured that (1) the ROIs did not overlap and (2) they lay within defined anatomical regions. The (ITS) ROI showed increased blood oxygen level dependent (BOLD) signal for words of decreasing spelling-sound consistency, and was spatially bounded by the inferior and middle temporal gyri. The (pFG) ROI was defined as an area showing increased BOLD signal with decreasing word frequency, restricted to not extend beyond the atlas definition of the fusiform gyrus. The (AG) ROI showed increased BOLD signal for reading words of increasing word frequency or imageability, and was masked to not extend beyond the atlas definition of the AG. The (pSTG) ROI showed increased BOLD signal with increasing response time (RT) for reading aloud, and the posterior MTG ROI (pMTG, bounded by the atlas definition of the MTG) showed increased BOLD signal for words of decreasing bigram frequency (Figure 1). These ROIs have previously been linked with different aspects of lexical retrieval: orthographic (pFG, e.g., Cohen et al., 2000; McCandliss et al., 2003; Binder et al., 2006; Vinckier et al., 2007), semantic (AG and ITS, e.g, Binder et al., 2009), and phonological processing (pSTG, e.g., Hickok and Poeppel, 2004, 2007; Graves et al., 2007, 2008; Gow, 2012; Price, 2012) as well as orthography-phonology mapping (pMTG, e.g., Jobard et al., 2003; Brambati et al., 2009; Richlan et al., 2009).

The timeseries of neural activation was separated into 4 sets per participant based on the characteristics of the words presented at each timepoint. The words were divided according to a 2 × 2 design with high and low levels of consistency and imageability, high and low levels of consistency and frequency, or high and low levels of frequency and imageability. Levels of each variable were defined as the upper and lower quartiles of the consistency, imageability, and frequency distributions from the complete stimulus dataset. There were on average 27.1 (*SD* = 10.4) trials in each cell of the 2 × 2 design table. Of these, only the trials on which participants made a correct response were considered for analysis. Responses were counted as incorrect if the participant stuttered, mispronounced the word, failed to respond, or responded with an RT more than 3 SDs from the group mean. The resulting timeseries of neural activation was aggregated across the 5 ROIs and 20 participants and was analyzed by condition using the IMaGES algorithm for effective connectivity (Ramsey et al., 2010; Tetrad software package http://www.phil.cmu.edu/projects/tetrad/). Candidate directed acyclic graphs were obtained using IMaGES search with a penalty discount optimized to find the first non-triangular configuration of connections. Next, connection directions were specified using the LOFS algorithm. LOFS belongs to a family of algorithms that exploit the fact that “the residuals of the correct linear model with independent non-Gaussian sources of error will be less Gaussian than the residuals of any incorrect model” (Ramsey et al., 2011, p. 4). This property of linear models is used in LOFS together with a non-Gaussianity (NG) measure to orient the effective connections. In our analysis we used the Anderson-Darling test of NG (Anderson and Darling, 1952). Model goodness of fit to each dataset was estimated using structural equation parametric modeling with a regression optimizer.

### Individual differences in effective connectivity

Follow-up analyses, aimed at better understanding the effective connectivity from AG to ITS that emerged only in the low-frequency, high-imageability word condition (see below), were performed in terms of the association of individual performance parameters with connection strengths. As this was the only connection that deviated from the stable network structure, which emerged across all conditions, we examined it closely. We found that the strength of this connection varied across participants. We tested for correlations between AG→ITS connection strength and mean RT for each participant and for correlations with individual effects of the main stimulus parameters of interest. These analyses were performed separately because including RT in the same multiple linear regression analysis with effects of stimulus properties defined in terms of their effect on RT would have resulted in an over-determined model. RT for in-scanner responses was calculated as the time from stimulus onset to response onset (for details see Graves et al., 2010). The behavioral effects of stimulus parameters were derived from a regression analysis performed separately on each participant, with RT as the dependent variable. RT was analyzed using multiple linear regression with the following six explanatory variables: length in letters, word frequency, consistency, imageability, the multiplicative interaction of word frequency, and consistency, and the multiplicative interaction of consistency and imageability. Values for these variables were mean-centered to avoid any multicollinearity that could result from inclusion of interaction terms (Kutner et al., 2005). This analysis resulted in for these variables were mean-centered to avoid -weights for each variable in each participant. The β-weights for consistency, imageability, and word frequency were then tested for correlation with AG→ITS connection strength across participants.

## Results

### The reading network

**Figure 2:**
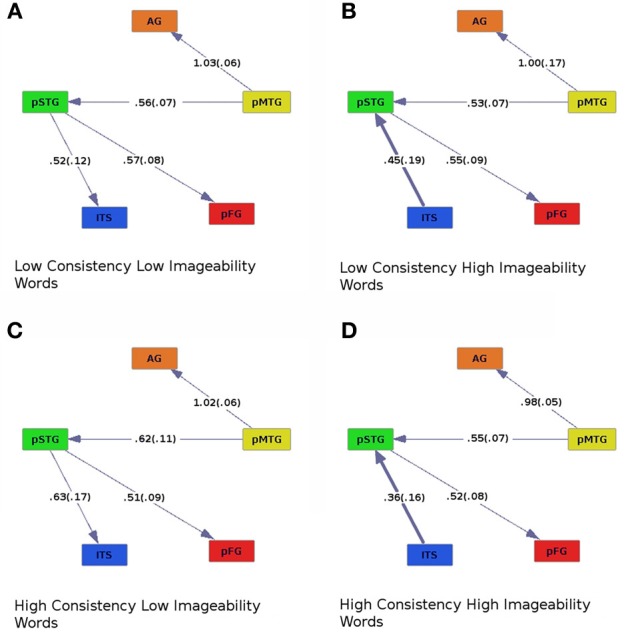
**Effective connectivity results for high (C,D) and low (A,B) levels of spelling-sound consistency crossed with high (B,D) and low (A,C) levels of imageability.** Colors correspond to Figure 1. Numbers along connections represent model regression fit coefficients averaged across participants, with standard errors in parentheses.

**Figure 3:**
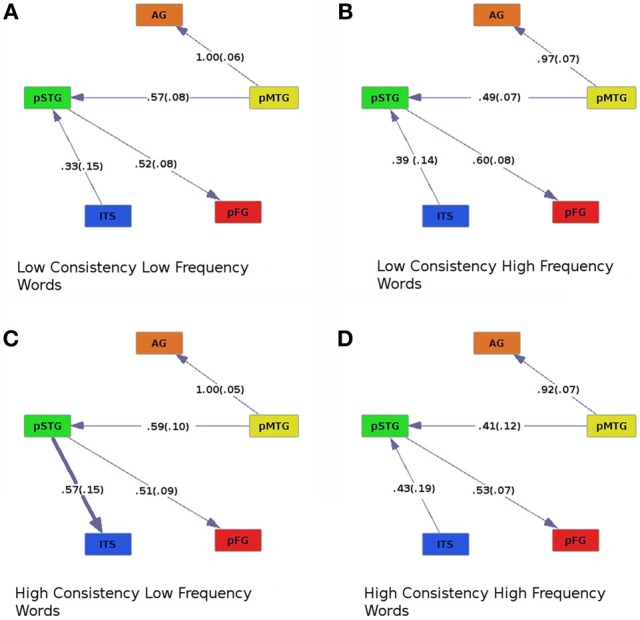
**Effective connectivity results for high (C,D) and low (A,B) levels of consistency crossed with high (B,D) and low (A,C) levels of word frequency.** Colors are as in previous figures, and numbers are as in Figure 2.

**Figure 4:**
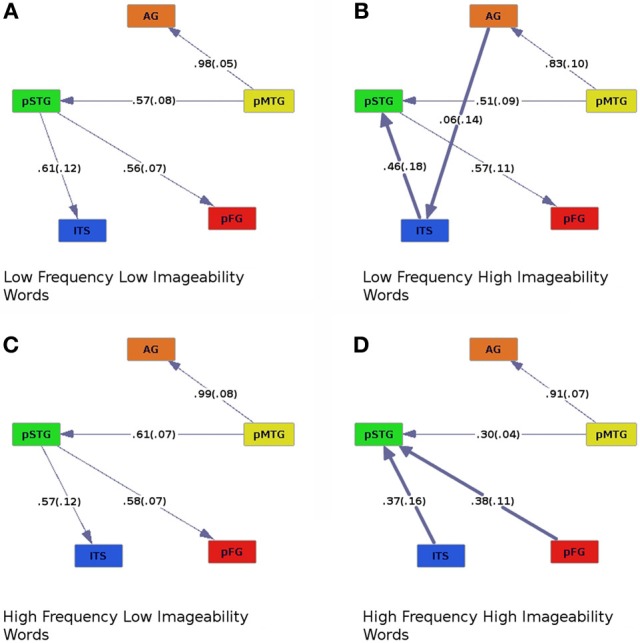
**Effective connectivity results for high (C,D) and low (A,B) levels of word frequency crossed with high (B,D) and low (A,C) levels of imageability.** Colors and numbers are as in previous figures.

Graphs of effective connectivity revealed a network of areas with some stable components, such as connections between AG and pMTG and between pMTG and pSTG, and components that varied depending on the nature of the stimuli. For example, the direction of connectivity between pSTG and ITS varied as a function of imageability. When imageability was high, ITS was driving the activity in pSTG, and when imageability was low, the pattern of effective connectivity was reversed (pSTG provided input to ITS, Figures 2, 4, where bold lines represent connections that differ across conditions). This connection was also modulated by frequency and consistency, with input from pSTG to ITS occurring only for words of high consistency and low frequency (Figure 3C). The connection between pSTG and pFG was more stable across conditions with only one reversal: for high-frequency high-imageability words only, pFG drove activity in the pSTG (Figure 4D). One additional connection, from the AG to ITS, was found for low-frequency and high-imageability words (Figure 4B). The strength of this connection had a direct relationship with performance on reading aloud, as described further below.

### Effects of consistency and imageability

Across all levels of consistency and imageability (Figure 2), activity in the pMTG, an area thought to be involved in orth→phon conversion (Jobard et al., 2003; Sandak et al., 2004; Brambati et al., 2009; Richlan et al., 2009; Graves et al., 2010), was found to influence activity in the AG, an area implicated in processing semantics (Binder et al., 2009), and pSTG, an area involved in processing phonology (Hickok and Poeppel, 2004, 2007; Graves et al., 2007, 2008; Gow, 2012; Price, 2012). Similarly, across all conditions, pSTG influenced activity in the pFG, an area thought to be involved in orthographic processing (Mechelli et al., 2000; Binder et al., 2006; Vinckier et al., 2007), and possibly some aspects of phonological processing (Hillis et al., 2005; Graves et al., 2010; Cattinelli et al., 2013; Mano et al., 2013). The effective connectivity graphs revealed a main effect of imageability. For high-imageability words, ITS, implicated in processing lexical semantics, drove activity in the pSTG, a phonological area (Figures 2B,D). The direction of effective connectivity between these areas was reversed for low-imageability words (pSTG provided input to ITS; Figures 2A,C). When regression coefficients were entered into an ANOVA with consistency (high or low), imageability (high or low), and connection (ITS to pSTG, pMTG to AG, pMTG to pSTG, and pSTG to pFG) as within-subjects factors, a main effect of connection [*F*_connect (3, 57)_ = 8.19, *p* < 0.001] and a marginal interaction between consistency and imageability was found [*F*_con × imag (1, 19)_ = 4.33, *p* = 0.051]. For high-imageability words, ITS provided stronger input to pSTG for low-consistency than for high-consistency words (Figures 2B,D).

### Effects of consistency and frequency

The effective connectivity graphs (Figure 3) revealed the same set of causal connections between pMTG, AG, STG, and pFG, as in the analysis of consistency and imageability. That is, pMTG modulated activity in AG and pSTG, which then influenced activity in the pFG. There was also an interaction between consistency and frequency, driven by a difference in the connectivity of ITS and pSTG. The pSTG drove activity in ITS only when participants read words of high-consistency and low-frequency (Figure 3C), otherwise pSTG drove activity in ITS. The interaction between consistency and frequency also influenced the strengths of causal connections across the ROIs. This was revealed by an ANOVA on regression coefficients for each participant, with consistency (high or low), frequency (high or low), and connection (ITS to pSTG, pMTG to AG, pMTG to pSTG, and pSTG to pFG) as within-subjects factors [*F*_con × freq (1, 19)_ = 4.64, *p* < 0.05]. There was also a main effect of connection [*F*_connect (3, 57)_ = 7.72, *p* < 0.001], and an interaction between consistency and connection [*F*_con × connect (3, 57)_ = 2.85, *p* < 0.05]. Connection strength was larger when pSTG modulated ITS in the high-consistency, low-frequency condition (Figure 3C) than when ITS modulated pSTG in all other conditions.

### Effects of frequency and imageability

Similar modulation by pMTG of the AG and pSTG was found as in the other analyses. Activity in pSTG modulated pFG in all but one case: When participants read words of high-frequency and high-imageability, this connection was reversed (Figure 4D). A main effect of imageability was observed, with ITS modulating pSTG for high-imageability words regardless of frequency, whereas the direction of effective connectivity between these regions was reversed for low-imageability words (compare Figures 4A–D). A main effect of imageability was found when regression coefficients were entered into an ANOVA with frequency (high or low), imageability (high or low), and connection (ITS to pSTG, pMTG to AG, pMTG to pSTG, and pSTG to pFG) as within-subjects factors [*F*_imag (1, 19)_ = 7.70, *p* < 0.05]. A main effect of connection was also observed [*F*_connect (3, 57)_ = 6.82, *p* < 0.005]. A three-way interaction between frequency, imageability and connection was also found, *F*_freq × imag × connect (1, 19)_ = 2.96, *p* < 0.05. Connection strength differed for high- compared to low-imageability words, such that connections from ITS to pSTG (high-imageability condition, Figures 4B,D) showed lower coefficients than connections from pSTG to ITS (low-imageability condition, Figures 4A,C). In addition, for high- and low-frequency words with low imageability (Figures 4A,C), the connection from pMTG to pSTG was stronger than for high-frequency words with high imageability (Figure 4D). An additional connection was found in the graph for low-frequency, high-imageability words, where AG modulated activation of ITS. This connection was found in no other condition. Following the direction of effective connectivity in this condition reveals an apparent cascade of activation as follows: AG→ITS→pSTG→pFG.

### Individual differences in effective connectivity

**Figure 5:**
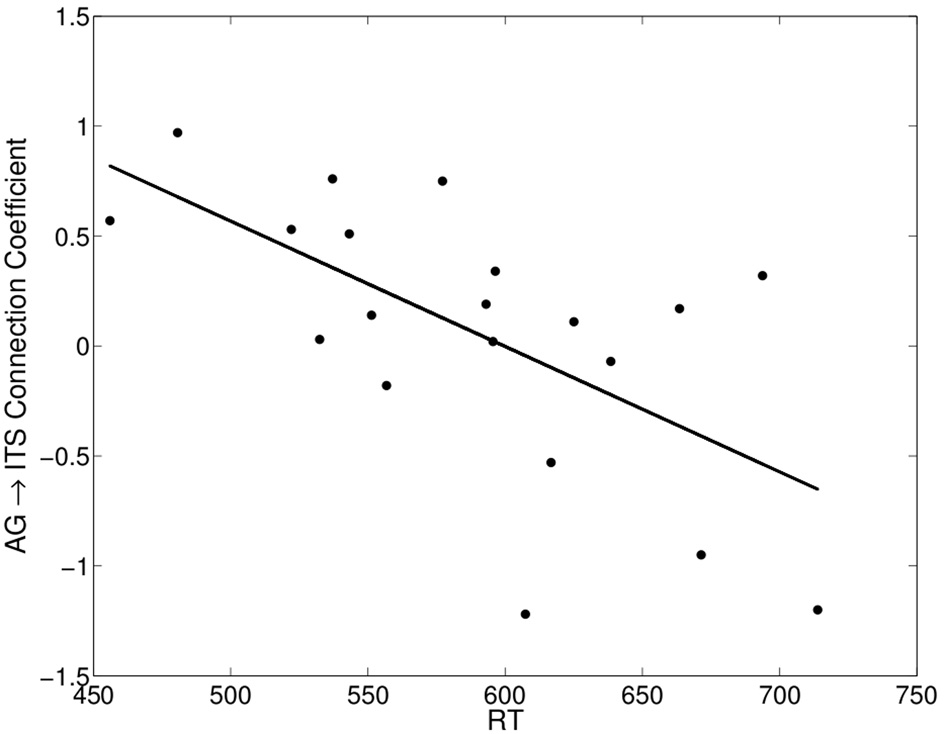
**Significant negative correlation between each participant's mean RT on correct reading aloud trials and the angular gyrus→inferior temporal sulcus connection coefficient (group connection shown in Figure 4B)**.

**Figure 6:**
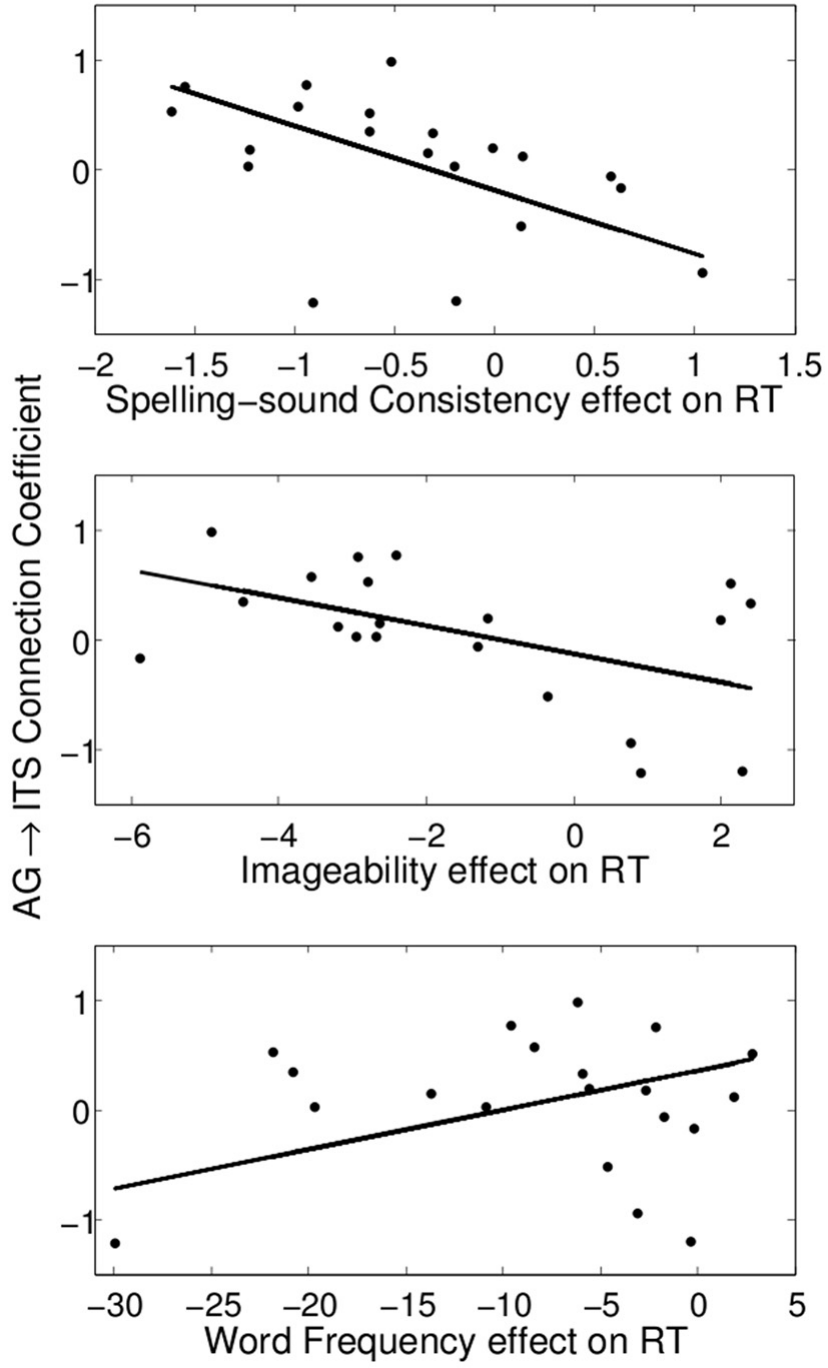
**Significant partial correlations between the consistency, imageability, and frequency effects on reading aloud RT and the angular gyrus→inferior temporal sulcus connection coefficient**.

The values of the regression coefficients for the AG→ITS connection varied considerably across participants, with a maximum strength of 0.97 and a minimum of −1.22. This was associated with individual variability in average RT on correct trials, such that stronger excitatory connectivity corresponded to faster RTs, *r* = −0.63, *t*_(18)_ = −3.40, *p* < 0.005 (Figure 5). We also examined the relationship between the strength of this connection and individual differences in how much the other factors being considered here—word frequency, consistency, and imageability—affected RT. A multiple linear regression model showed that all three of these variables contributed to predicting the AG→ITS connection strength, *F*_regression (3, 16)_ = 8.52, *p* < 0.005, and their joint effect explained 62% of the variance in the strength of the AG→ITS connection. The x-axis in Figure 6 represents β -weights for each factor, and each point is the effect for an individual participant. To use imageability as an example, individuals with negative values showed faster responses (lower RT) for higher imageabiltiy words, and the opposite was true for those with positive values. A decrease in the imageability [β = −0.53, *t*_(16)_ = 3.33, *p* < 0.005] and consistency [β = −0.69, *t*_(16)_ = 3.89, *p* < 0.005] β-weights, showing faster RT for high-imageability and high-consistency words, predicted a corresponding increase in AG→ITS connectivity. Conversely, an increase in word frequency effect values [*t*_(16)_ = 2.87, *p* < 0.05] predicted an increase in AG→ITS connectivity (Figure 6). The latter effect was unexpected and likely driven by a single outlier. With the apparent outlier removed from the analysis, the results revealed that the imageability and consistency effects remained significant, [β = −0.46, *t*_(15)_ = 2.48, *p* < 0.05; β = −0.71, *t*_(15)_ = 3.66, *p* < 0.005, respectively], but the frequency effect did not [β = 0.26, *t*_(15)_ = 1.22, *p* = 0.24].

## Discussion

In this study we analyzed effective connectivity among 5 ROIs previously shown to support different information processing components of reading aloud, including orthographic processing, orth-phon conversion, semantic access, and phonological processing. We investigated these components by varying levels of spelling-sound consistency, word frequency, and imageability. Following connectionist models of reading, we predicted that semantics would modulate lexical processing, especially under conditions when word imageability was high and word consistency was low, or when orth-phon mapping was not straightforward, such as in the case when low consistency was coupled with low word frequency.

Consistent with our predictions, effective connectivity analyses suggested that semantic access is an important component of reading aloud. Connectivity of ITS, an area previously implicated in processing word meanings (e.g., Binder et al., 2009; Graves et al., 2010; Whitney et al., 2011), and pSTG, a region involved in computing phonology (e.g., Hickok and Poeppel, 2004, 2007; Graves et al., 2007, 2008; Price, 2012), varied as a function of orthographic, phonological, and semantic characteristics of the stimulus words.

Our results are in line with accounts of reading aloud where phonology is computed interactively from semantic and orth-phon projections. The central role for ITS in mapping from semantics to phonology in our analysis is consistent with the Graves et al. (2010) findings, which showed that activity in this region was negatively correlated with spelling-sound consistency. Low-consistency words may invoke competing phonological codes and are the most likely to benefit from computational solutions that use semantic information to separate inconsistent words from their consistent word competitors. This assumption is in line with findings showing a clear link between activity in ITS/anterior MTG and single-word semantics (e.g., Jobard et al., 2003; Hickok and Poeppel, 2004; Binder et al., 2009; Whitney et al., 2011).

The role of AG, another semantic area (e.g., Binder et al., 1997, 2005, 2009; Price and Mechelli, 2005; Mechelli et al., 2007; Binder and Desai, 2011; Price, 2012), seemed to be distinct from that of the ITS. In our analyses, AG received feed-forward projections from pMTG, an area thought to be involved in mapping from spelling to sound (e.g., Jobard et al., 2003; Sandak et al., 2004), and their connectivity remained unchanged across all conditions. This pattern may reflect a more general activation of semantic and associative information in the AG (Graves et al., 2012). Furthermore, a closer look at the connectivity between AG and ITS revealed considerable individual variation in the strength of this connection related to participants' performance on the task, suggesting that AG engagement may be modulated by reading ability and dominant modes of processing by individual readers.

Regarding the cognitive models of reading, it is important to acknowledge that both the connectionist and dual-route models are incomplete with respect to relevant brain data. Indeed, the fact that the Taylor et al. (2013) meta-analysis explicitly addressed the role of effort in interpreting brain activations represents an innovative departure from the models they considered. Although connectionist reading models do offer specific, quantitative accounts of how differences in division of labor across reading pathways arise as a result of differences in stimulus properties, neither model deals with more domain-general issues such as how responses are selected or how working memory demands may be greater for some conditions than others. No doubt further exploring such issues will be an important direction for future studies.

Other models of functional and effective connectivity have also been described in the literature. In a finding largely consistent with the present study, Segal and Petrides (2013) reported that AG and its connectivity patterns play a central role in reading. They showed that functional connectivity between AG and MTG, STG, FG and IFG increased during reading words relative to viewing pictures. Similarly, Levy et al. (2009) used dynamic causal modeling (DCM) to model *effective* connectivity among brain regions. They showed increased connectivity between middle occipital cortex, posterior ventral occipitotemporal cortex, and left parietal lobe (white matter underlying the intraparietal sulcus) during reading word and pseudoword stimuli. Pseudowords and words differed primarily in that pseudowords had an intermediate connection with ventral occipitotemporal cortex, between middle occipital and parietal cortex, whereas effective connectivity for words did not include this ventral occipitotemporal region. Moreover, the strength of the connection from left middle occipital to left parietal cortices was correlated with several measures of reading performance. Overall, the Levy et al. study was similar to the current one in that it examined effective connectivity and individual differences in the neural reading network. However, several methodological differences (e.g., in tasks, contrasts, and analyses) may have contributed to the lack of overlap in results between the studies. Multiple word reading pathways were also reported in a DCM analysis by Richardson et al. (2011). They identified three pathways connecting an occipital visual area with temporal phonological and semantic areas. Two of the pathways traversed the ventral occipito-temporal cortex going either directly to anterior superior temporal sulcus (STS) or indirectly via posterior STS. The third pathway connected occipital visual cortex with posterior and anterior STS. Even though the Richardson et al. (2011) model did not include AG, because this area was not activated consistently across participants as a function of their contrast of interest, they showed a pattern of connectivity similar to ours where regions subserving orthographic and phonological processing are connected with regions supporting semantic processing (aSTS). We did not examine the role of aSTS in reading, however, a pathway going from occipito-temporal cortex to aSTS would necessarily pass through ITS, an ROI used in our analysis. This highlights the underlying similarity between our effective connectivity models and that of Richardson et al. On the other hand, the heterogeneity across models may stem from different cognitive processing models assumed by different groups, as well as from limitations on the number of ROIs, connections, and connection directions imposed by the DCM approach. Unlike DCM, the IMaGES approach used here does not incorporate biophysical assumptions. Instead, it offers a computationally tractable solution for discovering (rather than pre-specifying) connections and their directions among numerous ROIs (Ramsey et al., 2010). One promising avenue of future research might be to take multiple network-level neural models of reading that incorporate different cognitive processing assumptions and test them within the same effective connectivity framework such as IMaGES.

Overall, we take the current results to support the single-process connectionist account, with the caveat that this study is a preliminary investigation into the patterns of effective connectivity supporting reading aloud. The continuous design of the dataset we analyzed here was not optimized for the factorial analysis employed, and future studies are planned to address this limitation. Nevertheless, we found several qualitative and quantitative shifts in the directions of connectivity when contrasting high and low levels of spelling-sound consistency, imageability, and word frequency that paralleled independent predictions from connectionist models of reading aloud.

### Effects of consistency and imageability

The comparison between high- and low-imageability words revealed a main effect of this variable on patterns of effective connectivity. Regardless of consistency, for highly imageable words the ITS exerted a causal influence on pSTG (Figures 2B,D), whereas less imageable words showed the opposite pattern (pSTG→ITS, Figures 2A,C). One way to distinguish the two classes of words is to hypothesize that high-imageability words have more salient meanings, operationalized as the number of defining semantic features (e.g., Plaut and Shallice, 1993). When the task is to read aloud as quickly and accurately as possible, the reading system uses whatever information is at its disposal for each word. In the case of highly imageable words, semantic information is particularly salient, and several kinds of semantic information, including imageability, have been shown to facilitate reading aloud (Strain et al., 1995; Hino and Lupker, 1996; Lichacz et al., 1999; Strain and Herdman, 1999; Hino et al., 2002; Shibahara et al., 2003; Balota et al., 2004; Rodd, 2004; Woollams, 2005; Yap et al., 2012). Here we have shown that such facilitation by semantics may be neurally instantiated as an area associated with semantics (ITS) exerting a causal influence on an area associated with phonology (pSTG).

### Effects of consistency and frequency

In the analysis of the effects of consistency and frequency, pMTG was found to influence pSTG and this pattern was consistent across all conditions. In contrast, connectivity of ITS and pSTG varied. For example, we observed an interaction of word consistency and frequency, such that ITS influenced activity in pSTG for high-frequency words, regardless of consistency, and for low frequency words of low consistency (Figures 3A,B, and D). The opposite pattern, effective connectivity from pSTG to ITS, was found for high-consistency, low-frequency words (Figure 3C). Thus, we see evidence for the predicted switch between a lexical semantic processing area exerting influence on a phonological processing area for low-consistency words and the opposite pattern for high-consistency words, but only for words of low frequency. This may be a neural instantiation of a behavioral pattern. Psycholinguistic studies typically report an interaction between consistency and frequency, such that low-consistency words elicit longer RTs than high-consistency words, particularly if they are also of low frequency (Seidenberg et al., 1984; Seidenberg, 1985; Waters and Seidenberg, 1985; Taraban and McClelland, 1987; Andrews, 1992). Similarly, patients with surface dyslexia due to semantic dementia typically commit errors when reading aloud low-consistency words, especially when they are of low frequency (e.g., “sew” pronounced as “sue”; Patterson and Hodges, 1992; Woollams et al., 2007). Such an interaction is also inherent in the single-process model, as shown by a derived analytic solution describing the output state of a phoneme unit that should be activated for a given input word (equation 17 in Plaut et al., 1996). The activation state of the unit depends on input from the semantic and phonological pathways. This equation is a formalization of the pattern seen for word frequency and consistency. When the value of one variable is low, the value of the other variable matters more. This is paralleled in our analyses of effective connectivity, where consistency exerts an effect in the expected direction for lexical semantic and phonological areas, but only for low-frequency words. Why we did not see the complementary interaction of an enhanced effect of word frequency for low-consistency words is unclear. In addition, the equation discussed above from Plaut et al. (1996) predicts there would be little or no effect of word frequency for high-consistency words, yet our analyses did reveal frequency-related differences within the high-consistency condition (Figures 3C,D). Future work will be aimed at clarifying these results by exploring the role of imageability in producing these patterns of effective connectivity for words of high and low consistency and frequency. In the present study, insufficient numbers of trials were available to explore this three-way interaction. As noted above, we are currently planning a follow-up functional neuroimaging study of reading aloud in which the stimulus parameters of interest will be manipulated factorially (rather than continuously, as in the current study), and we expect new results to shed additional light on network-level neural systems that may correspond to network-level models of reading aloud.

### Effects of frequency and imageability

In the frequency by imageability analysis, we replicated the main effect of imageability obtained in the consistency by imageability analysis (Figure 2). Specifically, ITS drove activity in pSTG when word imageability ratings were high (Figures 4B,D), and the effective connectivity direction was reversed when imageability was low (Figures 4A,C). As described above, this pattern of results is interpreted as reflecting a neural correlate of the contribution of semantics to mapping from orthography to phonology.

In addition, reading high-frequency words of high-imageability also resulted in modulation of pSTG activity by pFG. The pattern of effective connectivity obtained in this condition suggests that when both word frequency and imageability are high the input from regions processing semantics (ITS) and regions subserving orthography-phonology mapping (pFG) converges on regions computing phonology (pSTG). This suggests that the highly efficient reading of these words is achieved by the strong convergence of multiple streams of relevant information.

For words of low frequency high imageability, an additional effective connection emerged from AG to ITS (Figure 4B). This is the only condition in which both semantic ROIs were effectively connected to each other, and it is the condition in which semantic information would be both available and beneficial to reading aloud. Importantly, the connectivity between these areas was associated with individual differences in performance measures. Stronger AG→ITS connections were associated with faster responses to high-consistency words, and faster responses to high-imageability words (Figure 6). In addition, stronger AG→ITS connections were unexpectedly associated with faster responses to low-frequency words. However, this latter effect seems to have been driven by a single outlier (see below). The strength of AG→ITS connectivity also facilitated RT on correct trials, pointing to a more general role of connectivity between these areas in reading performance. AG has often been associated with skilled reading. Decreased activity in AG (e.g., Shaywitz and Shaywitz, 2005) and pSTG (e.g., Simos et al., 2002; Shaywitz and Shaywitz, 2005; Maisog et al., 2008; Richlan et al., 2009), an area downstream from AG in Figure 4B, have been shown in studies comparing cases of developmental dyslexia to typical readers. Individuals with dyslexia (reading disability) typically exhibit poor phonological awareness, verbal working memory, and impaired lexical retrieval during visual word recognition (e.g., Shaywitz and Shaywitz, 2005). This is revealed by over-regularization of spelling-sound mapping or by an inability to read novel letter-sound combinations (Castles and Coltheart, 1993; Manis et al., 1996; Milne et al., 2003). Our analysis shows variations in AG→ITS connectivity associated with reading RT, suggesting that greater contributions from semantic areas are associated with better reading performance.

We see the current study as providing compelling evidence for the use of network-level effective connectivity analyses of functional neuroimaging data to reveal the information-processing dynamics of reading aloud. This approach also seems to offer clear evidence for modulation of division of labor by word properties of a sort not previously reported in the literature. However, we caution that the current study should be considered somewhat preliminary. The main reason for this (as mentioned above) is that, for purposes of the effective connectivity analyses, we applied a factorial approach to a continuous design study, thereby making it necessary to exclude intermediate-value observations. In addition, it seems that the word frequency result at the bottom of Figure 6 is being driven by an apparent outlier in the bottom left of the plot. We chose not to remove this data point because there was no obvious numerical criterion to apply that did not also reduce the fit of the overall multiple linear regression model. However, if data from this participant is simply removed, the apparent association between the behavioral word frequency effect and AG→ITS connection strength is no longer reliable, while the associations with consistency and imageability, though somewhat reduced in effect size, remain reliable in the same direction.

## Conclusion

In the present study we used effective connectivity analyses to advance the understanding of brain processes that support reading. Although it is often assumed that a network of brain regions must work together to accomplish complex cognitive tasks such as reading (Fiez and Petersen, 1998; Jobard et al., 2003; Indefrey and Levelt, 2004; Price, 2012), and that there is some degree of variability in the function of this network that corresponds to individual differences (Bolger et al., 2008; Seghier et al., 2008; Jobard et al., 2011; Welcome and Joanisse, 2012; Graves et al., submitted), the present study constitutes a detailed, data-driven evaluation of these assumptions. Here we showed that, under conditions predicted by connectionist models of reading aloud, semantics supports mapping from spelling to sound and that a range of individual variability exists in how much participants engage the semantic system. We also found that a core network of brain regions supports reading aloud, regardless of stimulus properties. Future work using a robust and flexible framework for effective connectivity analyses, such as IMaGES, applied across studies testing different cognitive models, may help clarify network-level neural accounts of reading.

### Conflict of interest statement

The authors declare that the research was conducted in the absence of any commercial or financial relationships that could be construed as a potential conflict of interest.

## Footnotes

^1^One of the reviewers pointed out that because Harm and Seidenberg (2004) showed essentially equivalent performance for inconsistent and consistent words along the orth→sem pathway, and most reading is presumably for meaning rather than reading aloud, the prediction that inconsistent words would engage semantics more than consistent words for reading aloud may not be obvious. However, as Harm and Seidenberg acknowledge, their focus on reading for meaning leaves open the possibility that in reading aloud, inconsistent words may benefit from semantics more than consistent words. Indeed, even if it also turned out to be the case in reading aloud that the orth→sem pathway were equivalently useful or activated by consistent and inconsistent words, this is only half of the semantic pathway in this task. For semantics to contribute to reading aloud, there must also be a mapping from semantics to phonology. The dynamics of the sem→phon path were not addressed in Harm and Seidenberg (2004), and it may be this part of the pathway that contributes differently for consistent and inconsistent words.
